# Effects of chopped CFRP fiber on mechanical properties of concrete

**DOI:** 10.1016/j.heliyon.2023.e13832

**Published:** 2023-02-17

**Authors:** Mand Kamal Askar, Lawend K. Askar, Yaman S.S. Al-Kamaki, Razaq Ferhadi

**Affiliations:** a Highways and Bridges Engineering, Technical College of Engineering, Duhok Polytechnic University (DPU), Kurdistan Region, Duhok, Iraq; b Civil Engineering Department, College of Engineering, University of Duhok (UoD), Kurdistan Region, Duhok, Iraq; c College of Engineering, The American University of Kurdistan (AUK), Kurdistan Region, Duhok, Iraq

**Keywords:** Concrete, Chopped CFRP, Concrete mechanical properties, CFRP fiber Addition, Low and normal strength concrete

## Abstract

Enhancing concrete's mechanical properties has become a prominent field in recent years. Numerous studies investigated the possibility of enhancing the mechanical properties of concrete by adding additive materials. Few studies investigated the effects of copped CFRP on the tensile strength of normal-strength concrete (NSC) and low-strength concrete (LSC). In this study, the effects of Chopped Carbon Fiber Reinforced Polymer (CCFRP) on the mechanical properties of LSC and NSC were investigated. The method of the study was experimentally investigating the effects of CCFRP on the mechanical properties of LSC and NSC. Different volume fractions (0%, 0.25%, 0.5%, and 0.75%) of chopped carbon fibers were added to the concrete mix for the 13 MPa and 28 MPa concrete grades, and five mix trials were conducted to achieve concrete with 13 MPa and 28 MPa. The ratios (1:1.5:2.5) for the normal strength mix and (1:2.6:4.1) for the low strength mix were chosen. Three tests were conducted to evaluate the effects of chopped CFRP on the mechanical properties of concrete: compressive strength, tensile strength, and flexural strength. A total of 120 pieces were cast, including 24 beams, 48 cubes, and 48 cylinders. The casted cubes were 15 × 15 × 15 cm and the cylinders were 15 cm in diameter and 30 cm in length. Prism beams with a 15 × 15 cm cross-section and a 56 cm length were tested under a single point load. The samples were tested at 7 and 28 days of age, and the sample density was recorded. The results revealed that adding 0.25% CCFRP increased the compressive strength of LSC from 9.5 MPa to 11.2 MPa which is about 10% enhancement and slightly affected the compressive strength of NSC by about 5%. On the other hand, adding 0.25% CCFRP to both LSC and NSC increased split tensile strength from 2.5 MPa to 3.6 MPa which is about 44% enhancement for NSC and 16.6% for LSC. Similar improvements were made in flexural strength of normal strength increased from 4.5 MPa to 5.4 MPa. Whereas the effects on LSC were unremarkable. As such, this study recommends 0.25% CCFRP fiber as the ideal dosage.

## 1 Introduction

Concrete is the most widely used construction material and the second most consumed substance in the world after water [1]. Concrete is an engineering material with a much higher compression strength than tensile strength. Consequently, concrete is regarded as a brittle material [2,3]. The provision of reinforcing steel is the standard approach taken to mitigate the effects of tensile stresses. However, under load, low tensile strength concrete leads to structural cracking and shorter structure life [4]. Adding fibers to concrete in general would act as crack inhibitors and substantially increase the tensile strength, cracking resistance, impact strength, wear and tear, fatigue resistance, and ductility of concrete [5]. Hence many studies in the last two decades investigated improving the tensile strength of concrete using additive materials such as fibers including glass fiber, polyethylene terephthalate (PET) fibers, and steel fibers [6]. PET fibers have some drawbacks in the production process, reduction, and workability [7]. Steel fibers also have some cons as claimed by Askar, Selman [8] such as workability, corrosion, and high cost. Glass fibers, on the other hand, have issues with a limited effect on concrete strength in comparison to their cost [9].

Steel fiber was the most popular concrete additive material over the last two decades. For the best results, the percentage of steel fiber concrete should be between 1% and 2%. Steel fiber acts as secondary reinforcement used in conjunction with traditional steel bars or prestressing strands as the main reinforcement. Several factors influence the effects of steel fiber on concrete strength, including type, shape, length, cross-section, strength, fiber content, steel fiber bond strength, matrix strength, mix design, and concrete mixture. Steel fiber has several advantages, including enhanced tensile strength, reduced initial cracking and flexural cracking, and increased ductility [10].

PET and PP are other additives to concrete that may enhance its strength. Many researchers have studied the effects of PET and PP on the mechanical properties of concrete in the last two decades [[11], [12], [13]]. This type of utilization is defined as adding PET or PP as fibers to the mix with a length ranges between 10 and 100 mm, width range between 1 and 10 mm, thickness range between 0.1 and 1.0 mm, and addition ratio range between 0.25% and 10% [14]. The fibers can be used also as polyester fiber with concrete mix. Length of the polyester fiber range between 3 mm and 40 mm with a 20–30 μm diameter. Adding 0.25% fiber polyester can increase compressive strength by 10–20%, and flexural strength by 5–15% with a reduction in split tensile strength by about 15–30% [[15], [16], [17], [18]].

Researchers are searching for novel construction materials to produce concrete with high tensile strength in order to decrease structural cracking and lengthen the life of structures. One of these novel materials is CCFRP. This material is newly provided by the CFRP manufacturer as it is chopped from various carbon fiber roving types. It offers superior properties such as high tensile strength, excellent corrosion and abrasion resistance, good conductivity, and resilience to high temperatures [19,20]. CCFRP is available in various lengths between 4 mm and 12 mm.

Kulikova, Sliusar [21], investigated the effects of chopped CFRP fibers on concrete with different length ranges between 3 and 9 mm. The results revealed a positive effect. Compression and bending strength were increased by 1.5% and 16% accordingly. Tensile strength was reduced slightly by about 4.5%.

Aziz and Taha [22] investigated the use of CCFRP to improve the compression and tensile properties of high-strength concrete. The result showed that adding 0.25%–0.5% CCFRP to the concrete mix enhances the flexural strength by about 23% and split tensile strength by about 25%, but compression strength improves by just about 2%. Li, Lee [23] claimed similar findings as test results demonstrated that adding 1% CCFRP improved compressive strength by 20%. This finding is agreed upon by Li, Li [24].

In contrast, Aziz and Taha [25] investigated the impact of chopped CFRP on the flexural behavior of high-strength concrete. The result indicated that the presence of CCFRP will increase the first crack load by 33% when 0.25% is added and by 200% when 0.5% is added with negligible improvement to the ultimate load. According to Mastali and Dalvand [26] adding 0.25% CCFRP led to the best result in terms of the first cracking load. Li, Lee [23] added 1% CCFRP to the concrete mix, and the flexural strength increased by 25%. Li, Li [24] investigated the effects of chopped CFRP on concrete flexural strength and the result showed similar findings.

Generally, adding natural and synthetic fibers to reinforced concrete structures can increase their toughness, durability, and ability to decrease permeability, shrinkage, and creep [27,28]. Such an additive can also prevent cracking brought on by drying shrinkage and plastic shrinkage. Additionally, certain fiber lengths could provide concrete additional impact resistance, abrasion resistance, and fracture resistance [28]. Li et al. [23] conducted tests on the free-fall impact behavior of concrete and mortar with different lengths of chopped fiber (6, 12, and 24 mm). The free-fall impact test results show that the 24 mm chopped fiber enhances their impact numbers more than the other lengths of fibers, and the benchmark samples.

In most cases, the microscopic morphology of the chopped carbon fibers in the concrete can be examined by scanning electron microscopy (SEM). Li et al. [29] observed a uniform dispersion of fibers leads to better cross-linking effects, which were found by the SEM. Seker et al. [30] carried out an SEM inspection in order to investigate the interface mechanism between fibers and mortar. SEM measurements reveal that during the mortar mixing process, chopped glass fibers were separated into individual fibers. However, carbon fibers can be seen as unique.

CCFRP is a novel additive fiber that demands additional investigation into the mechanical properties of not only high-strength concrete as presented in the literature but also NSC and LSC which is considered as the presented research novelty. Moreover, the main aim of the current work is to identify the optimum dosage of CCFRP fibers in LSC and NSC. Thus, the effects of CCFRP on the mechanical properties of NSC and LSC are examined and the optimum dosage of CCFRP in concrete is illustrated.

2. Mechanism of CCFRP

CCFRP is a novel construction material, which has been the subject of limited studies about its actual effects on the overall characteristics of concrete. In general, adding fiber to concrete results in an increase in the tensile strength of concrete. Similar to conventional concrete fibers, CCFRP fibers provide high resistance to plastic shrinkage cracking and drying shrinkage cracking, as well as maximum resistance to damage from heavy impact. The mechanism of crack formation is slightly altered by the presence of fibers. The bridging of fibers can help some tensile loads pass through the fissures. As a result, the tension in the concrete is caused by both bond stress and fiber bridging. Less bond stress is required to achieve the same cracking stress when the fibers' contribution is taken into account. Fibers are pulled out and the benefit from fiber bridging is reduced due to the breakdown of the link between the fibers and concrete under high stress [10,31].

The high tensile fibers emerge in the concrete matrix and begin a tendency to redistribute stresses evenly throughout the matrix and contribute to the post-cracking strength and restraining of the cracks in the concrete. This led to an increase in concrete tensile strength. This process requires extra water compared to concrete without fibers and results in a significant reduction in concrete workability. Adding fibers to concrete reduces concrete permeability and, thus, reduces the bleeding of water [10].

**Fig. 1:**
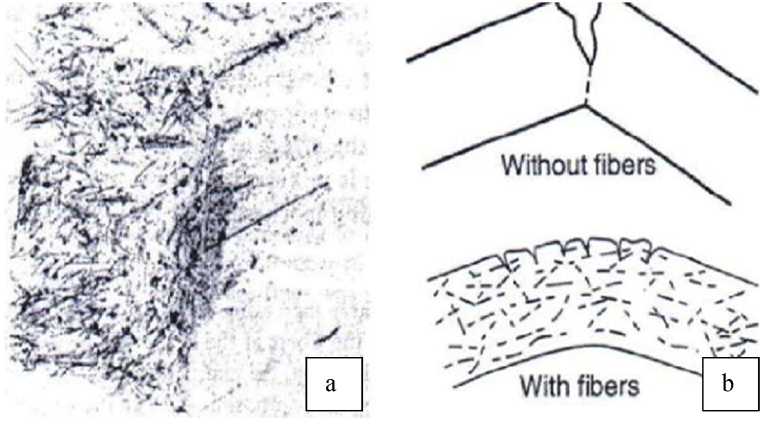
Concrete with and without fibers. a) concrete section contains fibers; b) concrete behavior with and without fibers [32].

Under load, fiber-reinforced concrete performs better than conventional concrete because the uniformly distributed and randomly aligned fibers improve the initial breaking load by improving the tensile strength of the concrete. This mechanism will also increase the flexural strength of concrete, however, the primary reason for adding fiber to concrete is not to add strength but to prevent cracking from drying shrinkage or plastic shrinkage, Fig. 1 [32].

## 2 Experimental work

All essential experimental work has been performed and illustrated in this publication.

### 2.1 Materials

The following materials were utilized for concrete production.

#### 2.1.1 Cement

In this research, OPC I-42.5 R cement was used, which was manufactured by the Lafarge factory-Iraq. The physical and chemical composition of cement studied and depicted in Table 1 according to ASTM C150 [33], BS EN 2013 [34], BS EN 2016 [35], IQS 1984 [36].

**Table 1:** Chemical and physical properties of cement.

Physical, mechanical, and chemical properties	Testing method	Limitation	Result
Loss on ignitions (%)	IQS472/1993	≤4.0	3.37
Non-Soluble Substances (%)		2.5 if C_3_ A≤3.5	0.72
Sulfate Content (as So_3_) (%)		2.8 if C_3_ A≥3.5	2.38
Tricalcium Aluminates (C_3_A) (%)		Not specified	4.09
Magnesium Oxide (as Mgo) (%)		≤5.0	3.75
Chloride Content (%)	BS EN 196–2/2013	≤0.1	0.02
Finesse (Blaine) (M^2^/kg)	IQS 198/1990	≥280	350
Initial Setting Time (minute)	BS EN 196–3/2016	≥45	160
Final Setting Time (hr)		≤10	3.30
Soundness(expansion) – Le chattel (mm)		≤10	0.0
Compressive strength is not less than (MN/m^2^) 2 d	BS EN 196–3/2016	≥20	25.7
Compressive strength is not less than (MN/m^2^) +28 d		≥45	52.0

#### 2.1.2 Fine aggregate

In this experiment, coarse-grained, naturally occurring clean river sand was collected from the Alkhazer quarry and employed as fine aggregate. Table 2 displays the results of the sieve analysis of fine aggregate. Sand gradation conforms to ASTM C136 [37], ASTM C33 [38], ASTM C127 [39], and IS-383 [40].

**Table 2:** Fine aggregate sieve analysis.

Sieve sizes mm	(Natural aggregate)		
	Accumulative remaining (gr)	remaining (%)	passing (%)
19		0.0	100.0
12.7		0.0	100.0
9.5		0.0	100.0
4.76	205.0	13.6	86.4
2.38	585.0	38.7	61.3
1.19	838.0	55.5	44.5
0.600	1002.0	66.4	33.6
0.300	1253.0	83.0	17.0
0.149	1420.0	94.0	6.0
0.074	1489.0	98.6	1.39
Fineness modulus			3.512
Dry weight (A)	1510.0		
Dry weight after washing	1491.0		
% Passing (A-B)/A*100	1.26		

#### 2.1.3 Coarse aggregate

As a coarse aggregate, crushed stone with a maximum size of 10 mm sourced from the Alkhazer quarry was utilized. Table 3 displays the actual grading obtained from sieve analysis in accordance with, ASTM C33-99a [38], C136 [37], ASTM C127 [39], and IS-383 [40].

**Table 3:** Coarse aggregate sieve analysis.

Sieve sizes mm	(Natural aggregate)		
	Accumulative remaining (gr)	remaining (%)	passing (%)
37.5	0.00	0.00	100
25.4	0.00	0.00	100
19	939	26.4	73.6
12.7	2776	78.0	22
9.5	3423	96.1	3.9
4.76	3559	99.9	0.1
Dry weight (A)			3561
Dry weight after washing			3559
% Passing (A-B)/A*100			0.06

#### 2.1.4 Water

Tap potable water at laboratory temperature was utilized to wash coarse aggregate, mix concrete, and cure samples throughout this investigation in accordance with ASTM C 1602.

#### 2.1.5 Chopped CFRP fiber

**Fig. 2:**
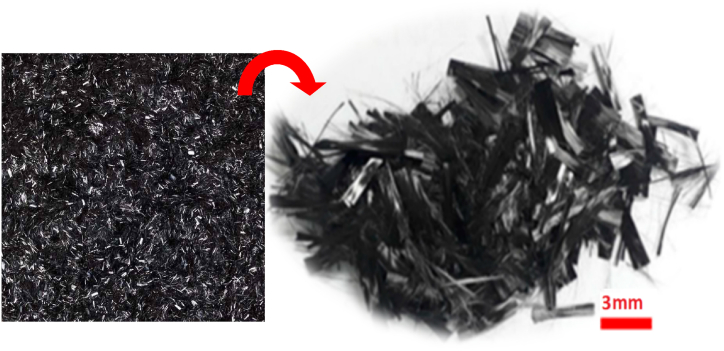
Chopped CFRP.

Chopped carbon fiber reinforced polymer fiber was used in this research. The fibers had a length of 3 mm and a tensile strength of 4.5 GPa Table 4. According to Mallick [41], short fibers are not considered discontinuous fibers because their length exceeds 1 mm. CCFRP has great conductivity, tensile strength, and corrosion resistance, and it comes in black color Fig. 2.

**Table 4:** CCFRP properties.

Properties	Value
Available length (mm)	*3, 4, 6, 8, 10, 12, 24
Color	Black
Ø (μm)	7–8
Density (g/cm^3^)	1.76
Tensile Strength (GPa)	4.5–4.9
Tensile Modulus (GPa)	220–240
Elongation	1.5%
Carbon content	≥95%
Carbon filament	12 k

*: CCFRP used length is 3 mm.

#### 2.1.6 Superplasticizer

To improve workability, a high range water-reducing admixture (superplasticizer) with the trade name Sika ViscoCrete 5930 was added to the mixtures. According to the manufacturer, the dose range for the blinder should be between 0.2% and 0.8% by weight. This form of admixture conforms to ASTM C494 [42]. The key characteristics of this superplasticizer are listed in Table 5**.**

**Table 5:** Specifications of superplasticizer.

Properties	Description
Appearance	Turbid liquid
Specific gravity	1.085 ± (0.005) g/cm^3^
Chloride content	Nil
pH value	4–6

### 2.2 Method of study

**Fig. 3:**
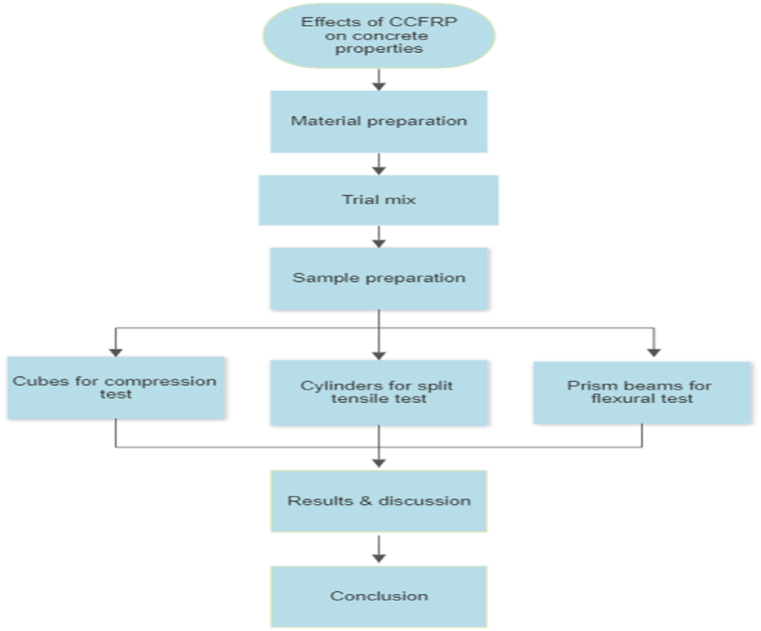
Flowchart explains the methods of the research.

The method of the study was preparing the required material to obtain two concrete grades using trial mix procedure. Then required samples were casted to do compression, split tensile and flexural tests. Result then discussed and a conclusion and recommendations were illustrated Fig. 3.

### 2.3 Concrete mix

Seven trail mixes were undertaken to obtain two different concrete grades of 13 and 28 MPa without CCFRP. For each mixture, a set of six 150 × 150 × 150 mm cubes were cast and tested after 28 days. The samples were cured at the laboratory temperature for 28 days. Two mixture proportions were selected as listed in Table 6. The selected mixes were based on the required concrete grades and the optimum workability achieved between the mixes. The quantities of materials shown in Table 6 correspond to one cubic meter. Then, the concrete mix of the selected mix design was poured into the mold in three layers, and each layer was manually tempted 36 times using a standard square rod made of steel with a rounded or bullet-shaped end (16 mm diameter and 60 mm in length) according to ASTM standards. Cylinders were compacted 25 times with round, and straight tamping steel rod (10 mm diameter × 305 mm long).

**Table 6:** Concrete trial mixes by weight.

Trials mix	Mix proportion	Cement	Fine aggregate	Coarse aggregate	w/c %	SP %	$f_{c}^{\prime }$ ***
1	1:3:6	1.763	5.289	10.578	0.61	–	23.1
2	1:3:6	1.763	5.289	10.578	0.61	1.6	23.2
3	1:3:6	1.763	5.289	10.578	0.61	1.4	18.8
4	1:3:6	1.763	5.289	10.578	0.61	2	22.2
5	1:2.6:3.2	3.4	8.84	13.94	0.613	–	27.85
6*	1:2.6:4.1	3.4	8.84	13.94	0.71	–	13.00
7**	1:1.5:2.5	5	7.5	12.5	0.5	–	28.16

*Adopted mix design as LSC, ** adopted mix design as NSC, *** $f_{c}^{\prime }$ is cylinder concrete strength at 28 days.

### 2.4 Sample preparation

**Fig. 4:**
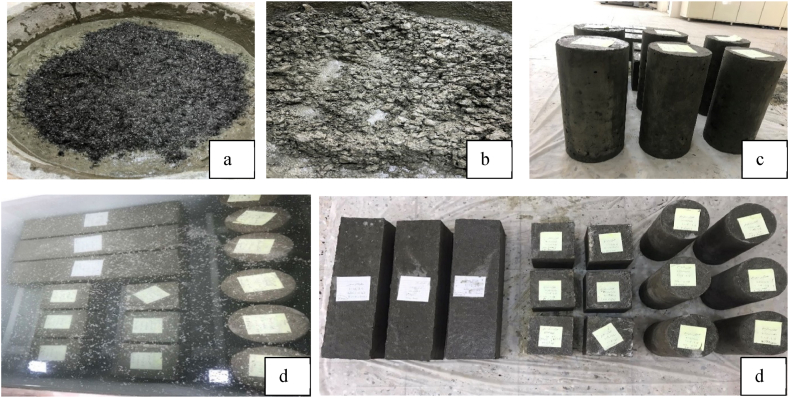
Sample preparation. a) mixing chopped CFRP fibers with concrete; b) concrete after adding fibers; c) samples at age of one day; d) curing; e) preparation samples for testing.

Three major tests were performed in this study: compression, split tensile, and flexural. Slump test and density were also recorded. A total of 48 cubes, 48 cylinders, and 24 prisms were cast in this study Table 7. The cubes were 15 × 15 × 15 cm in size, the cylinders were 15 cm in diameter and 30 cm in height, and the prisms were 15 × 15 cm in cross-section and 56 cm long. Three CCFRP fiber ratios were added to the mix 0.25%, 0.5%, and 0.75% different from the control samples. CCFRP is added to the dry mix followed by water. When CCFRP was added, the mix's workability dropped dramatically. To solve this issue, a superplasticizer (ViscoCrete 5930) was utilized to enhance workability. Samples were then cured in a water tank until the testing date Fig. 4. Half of the cubes and cylinders were tested at 7 days and the remaining half were tested at 28 days. The prisms were tested at 28 days.

**Table 7:** Sample design matrix.

Samples	Mix	Samples			Cement Kg	Fine aggregate Kg	Coarse aggregate Kg	W/C %	SP %	CCFRP %
		cube	cylinder	prism						
*CN 0%	1:1.5:2.5	6	6	3	43.924	65.886	109.81	0.5	0	0
CN 0.25%	1:1.5:2.5	6	6	3	43.924	65.886	109.81	0.5	0.2	0.25
CN 0.5%	1:1.5:2.5	6	6	3	43.924	65.886	109.81	0.5	2	0.5
CN 0.75%	1:1.5:2.5	6	6	3	43.924	65.886	109.81	0.5	2	0.75
CL 0%	1:2.6: 4.1	6	6	3	29	75.4	118.9	0.71	0	0
CL 0.25%	1:2.6: 4.1	6	6	3	29	75.4	118.9	0.71	0.7	0.25
CL 0.5%	1:2.6: 4.1	6	6	3	29	75.4	118.9	0.71	1.6	0.5
CL 0.75%	1:2.6: 4.1	6	6	3	29	75.4	118.9	0.71	2	0.75
	Sum	48	48	24						

*CN: normal strength concrete; CL: low strength concrete; the number after the letter represents the CCFRP ratio.

### 2.5 Bulk density test

The density of samples was measured and recorded to demonstrate any change in density caused by the addition of CCFRP. The density was found by dividing the mass of a cube by its volume.

In addition, the total density of the cubes was determined by measuring the mass of the cubes after pulling them out of the water tank and leaving them for 60 ± 5 min to allow the curing water to expel. Furthermore, each of the cube dimensions (Length, Width, and Height) was measured three times and the average was used in the calculation.

### 2.6 Compression test

**Fig. 5:**
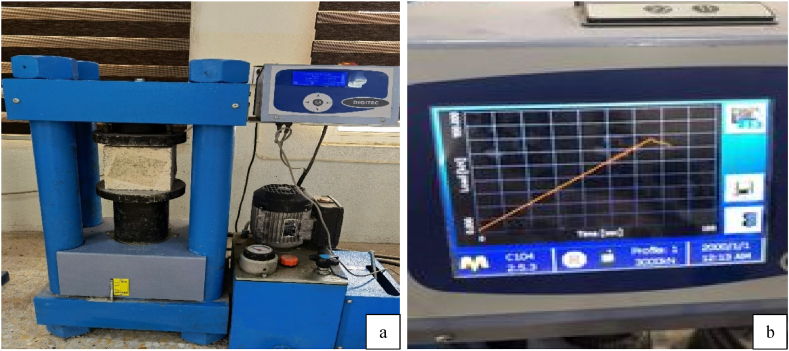
Samples under compressive strength test. a) sample during compression test; b) Ultra sonic machine.

This test was in accordance with BS 116–1983 [43], BS 12390–1:2000 [44]. Using 150 × 150 × 150 mm cubes, the compressive strength of concrete was determined. The experiment was carried out at two ages, precisely at 7, and 28 days. There were three specimens utilized for each test. The results of the tests were used to determine the mean value. A Matest compression machine with a capacity of 2000 kN was employed. Fig. 5 demonstrates that a uniform loading rate of 5 kN/s was applied until the cubes failed and the ultimate load was recorded. Compressive strength was found.

### 2.7 Split tensile test

**Fig. 6:**
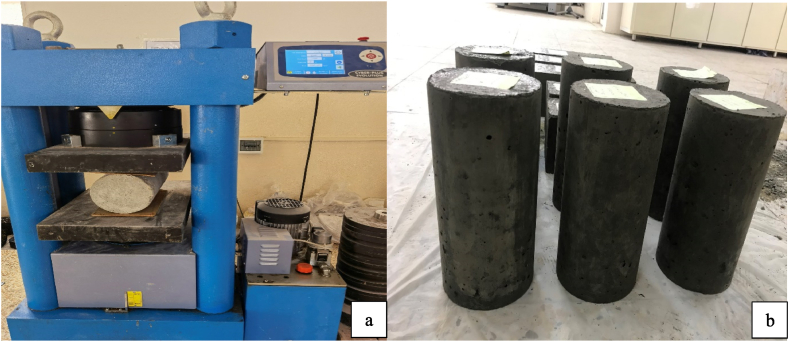
Samples under split tensile strength. a) sample under split tensile test; b) cylindrical samples to conduct split tensile test.

Split tensile strength testing was conducted in accordance with ASTM C 496 [45], with cylinder molds of 150 mm in diameter and 300 mm in height**.** Fig. 6 shows a 2000 kN Matest test machine with a load rate of 1 kN per second. Prior to testing, the specimens, surfaces were dried and horizontally placed on the machine plate, with strips added to avoid crushing concrete specimens at the bearing points. The split tensile strength was calculated.

### 2.8 Flexural test

**Fig. 7:**
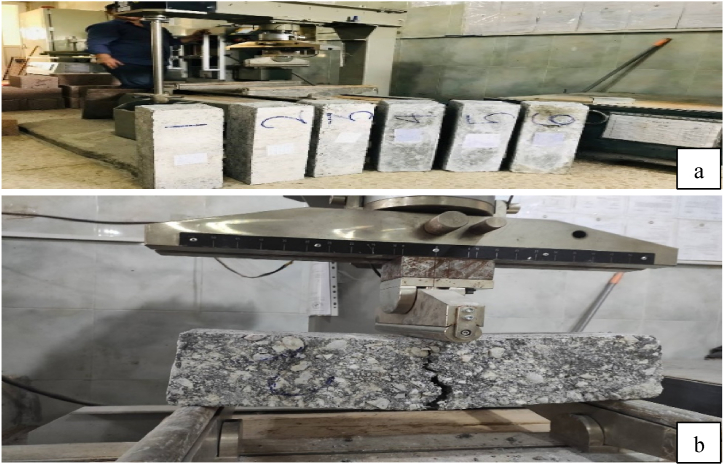
Samples under flexural test. a) prism samples prepared for testing; b) flexural test machine.

A flexural test was carried out in accordance with ASTM C 293 [46]. This study employed prism beams of 560 mm in length, 150 mm in width, and 150 mm in depth. Fig. 7 shows three-point loading with a load rate of 1 MPa/min. This test was carried out using a Matest testing equipment. The specimens were first surface dried after curing, and the loading and support sites were marked with a marker. The ultimate load was then measured, and the modulus of rupture was calculated.

## 3 Result and discussion

The results show that CCFRP enhances the strength properties of concrete when used in low dosage and degrades concrete strength when the dosage is increased. Table 8 and Table 9 show the test results for two grades of concrete: LSC and NSC. Slump test, density, compressive strength, split tensile strength, and flexural strength of concrete were all performed.

**Table 8:** Normal strength concrete test results.

CCFRP ratio	7days		28days			Density (kg/m^3^)	Slump test (mm)
	fc’ (MPa)	ft (MPa)	fc’ (MPa)	ft (MPa)	fr (MPa)		
0%	15.1	2.2	28.5	2.5	4.5	2425	190.0
0.25%	17.7	2.4	30.1	3.6	5.4	2416	190.0
0.50%	11.8	1.8	23.1	3.5	4.8	2350	30.0
0.75%	11.6	1.5	20.5	3.0	4.7	2331	10.0
Equations:	$fc^{\prime }=\frac{P}{A}$	$ft=\frac{2\times P}{\pi \times D\times L}$		$fr=\frac{3\times P\times L}{2\times b\times d^{2}}$		$Density=\frac{M}{V}$

**Table 9:** Low strength concrete test results.

CCFRP ratio	7days		28days			Density (kg/m^3^)	Slump test (mm)
	fc’ (MPa)	ft (MPa)	fc’ (MPa)	ft (MPa)	fr (MPa)		
0%	9.5	1.1	13.2	1.8	3.6	2379	140.0
0.25%	11.2	1.3	14.5	2.1	3.7	2349	40.0
0.50%	9.3	1.2	11.6	1.8	3.2	2292	20.0
0.75%	9.2	1.1	9.4	1.7	3.1	2291	5.0

### 3.1 Slump test

Despite the use of water reducer ViscoCrete 5930, the presence of CCFRP had a substantial impact on the slump test findings. When 0.5% CCFRP was added to LSC, the slump test value decreased from 140 mm to 20 mm. On the other hand, when CCFRP is introduced to regular-strength concrete, the slump decreases from 190 mm to 30 mm.

### 3.2 Density

The density of dried samples was measured and shown in Table 8, Table 9 for both LSC and NSC. When 0.75% CCFRP fiber was introduced, test results showed that it resulted in a modest loss in the concrete density of up to 4%.

### 3.3 Compressive strength

**Fig. 8:**
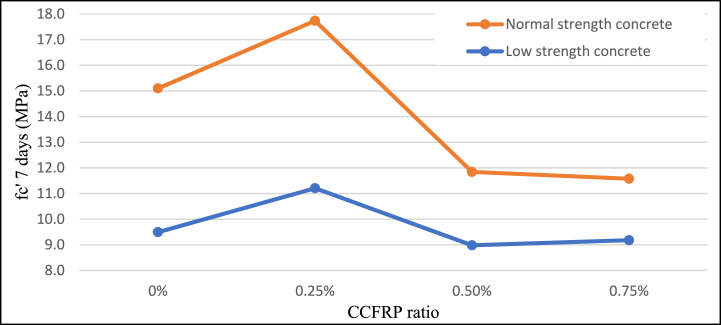
Compressive strength versus CCFRP ratio at age of 7 days.

**Fig. 9:**
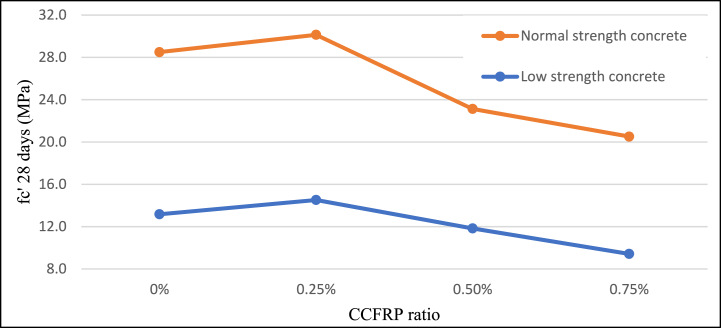
Compressive strength versus CCFRP ratio at age of 28 days.

**Fig. 10:**
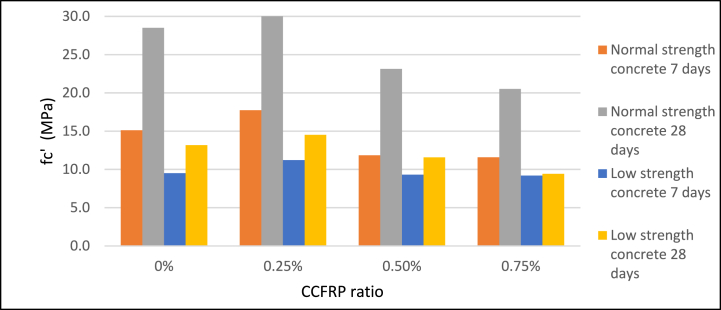
Compressive strength versus CCFRP ratio.

When a 0.25% CCFRP ratio was added to the mix, the compressive strength of 28-day samples of NSC and LSC rose by approximately 6% and 10%, respectively as agreed to Sbahieh, Tahir [47]. As the ratio of CCFRP increased, compressive strength decreased by approximately 20% for 0.5% CCFRP and 40% for 0.75% CCFRP. According to the 7-day age samples test results, the addition of 0.25% CCFRP significantly increased early-age strength, however, the addition of 0.5 and 0.75% CCFRP had a negative effect on the compressive strength of concrete Fig. 8**,** Fig. 9, and Fig. 10. Compared with normal additive fibers, CCFRP has a lower beneficial effect on the compressive strength of concrete.

### 3.4 Split tensile strength

**Fig. 11:**
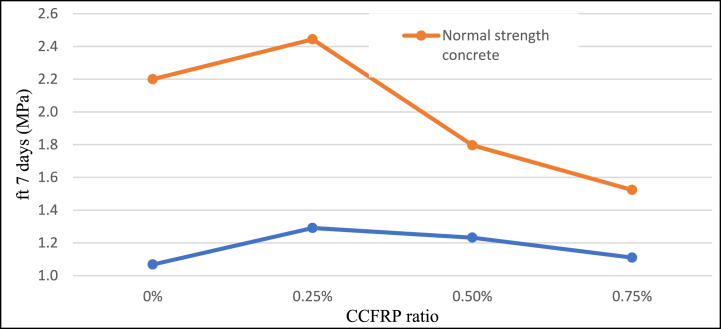
Split tensile strength versus CCFRP ratio at age of 7 days.

**Fig. 12:**
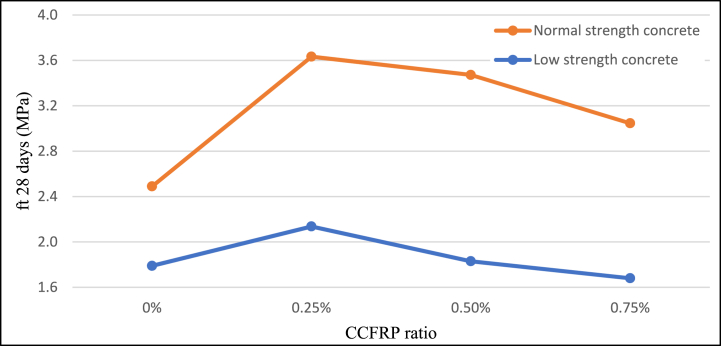
Split tensile strength versus CCFRP ratio at age of 28 days.

**Fig. 13:**
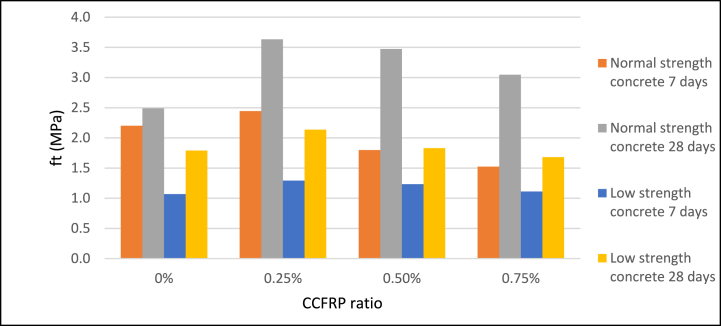
Split tensile strength versus CCFRP ratio.

Adding 0.25% CCFRP to the concrete mix increases split tensile strength by approximately 44% for normal strength samples tested at 28 days and by around 17% for LSC. The behavior was similar for 7 days-aged samples. This favorable effect of CCFRP becomes negative when the dosage of CCFRP is increased Fig. 11**,** Fig. 12, and Fig. 13. These results indicate that CCFRP highly improved the split tensile strength of concrete and this material can be a replacement material for traditional steel fiber additives.

### 3.5 Flexural strength

**Fig. 14:**
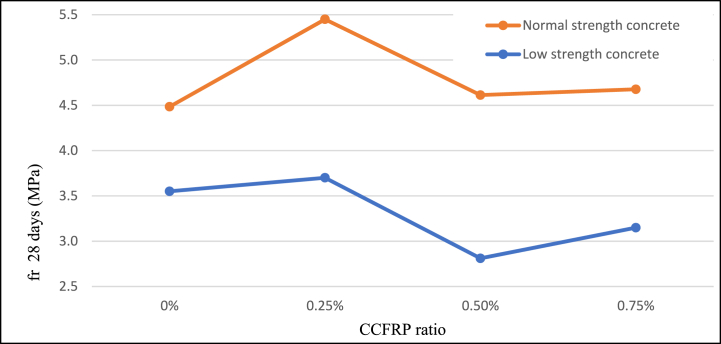
Flexural strength versus CCFRP ratio at age of 28 days.

Adding 0.25% CCFRP increases flexural strength by approximately 20% for normal-strength samples and by approximately 3% for low-strength samples. Increased CCFRP dosage resulted in a little enhancement in flexural strength for normal strength samples and negative effects with low strength samples Fig. 14. These results indicate that CCFRP has similar behavior to traditional additive fibers.

## 4 Evaluation of previous studies conducted on CCFRP in concrete

In this study, Table 10 illustrates and discusses in depth the findings of past research that examined the impact of using CCFRP as an additive material on concrete strength.

**Table 10:** Previous studies on utilizing CCFRP.

Author	Concrete strength (MPa)	CCFRP ratio	Length (mm)	CCFRP properties	Effects on concrete strength compared to control sample				
					$f_{c}^{\prime }$ %	$f_{t}$%	$f_{r}$%	Slump test %	Density %
Aziz and Taha [22]	60	0.25%	20	Ø = 7–8 μm $f_{u}$ = 2.84 GPa E = 235 GPa	1.60	4.07	23.76		
		0.50%			2.11	23.58	40.59		
	80	0.25%			1.98	5.38	23.77		
		0.50%			2.55	26.08	40.98		
	100	0.25%			2.65	5.71	20.90		
		0.50%			2.93	28.57	41.04		
Muley, Varpe [48]	50	0.25%	6	Ø = 7–8 μm $f_{u}$ = 4.3 GPa E = 230 GPa	8	13	13		
		0.50%			12.7	23.7	29.5		
		0.75%			22.8	31.6	36		
		1.00%			38.4	42.1	52		
Mastali and Dalvand [26]	40	0.25%	20	Ø = 7–8 μm $f_{u}$ = 3.55 GPa E = 235 GPa	1.3		1.3		
		0.75%			1.5		1.55		
		1.25%			1.7		1.7		
Ghanem and Bowling [49]	41	0.5%	6	Ø = 7–8 μm $f_{u}$ = 3.55 GPa E = 235 GPa	2	18	3	- 28	
		1.0%			13.6	43	5	- 43	
		1.5%			1.8	74	13	- 55	
		2.0%			- 3	132	13.8	- 85	
AL-Ridha, Abuzaid [50]	25	0.2%	6	Ø = 7–8 μm $f_{u}$ = 3.45 GPa E = 230 GPa	11.6	43			
			12		23	68.8			
Li, Lee [23]	32	1.00%	6	Ø = 7 μm $f_{u}$ = 4.9 GPa E = 250 GPa	22.2				
		1.00%	12		14.0				
		1.00%	24		11.3				
Li, Li [24]	22	5.00%	10	Ø = 7 μm $f_{u}$ = 4.9 GPa E = 250 GPa	31.0		11.2		
		10.0%			37.6		39.6		
		15.0%			30.0		27.7		
Current study	13	0.25%	3	Ø = 7–10 μm $f_{u}$ = 4.6 GPa E = 230 GPa	9.8	44	2.7	−71	- 1
		0.50%			−12.1	40	−11.1	- 86	- 4
		0.75%			−28.8	20	−13.9	−96	- 4
	28	0.25%			5.6	16.6	20	0	0
		0.50%			−18.9	1	6.6	- 84	- 3
		0.75%			−28.1	−5.5	4.4	- 95	- 4

**Fig. 15:**
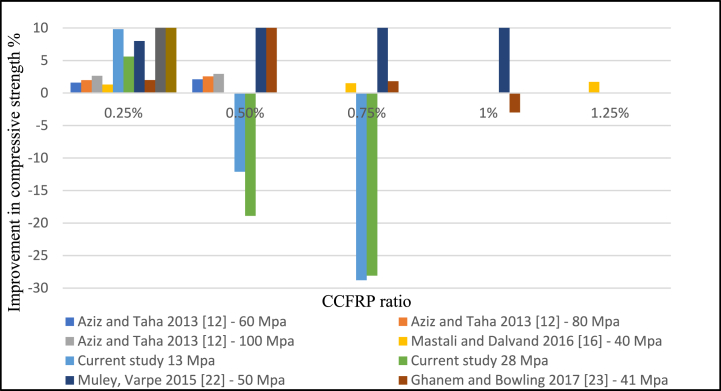
Compressive strength versus CCFRP ratio.

**Fig. 16:**
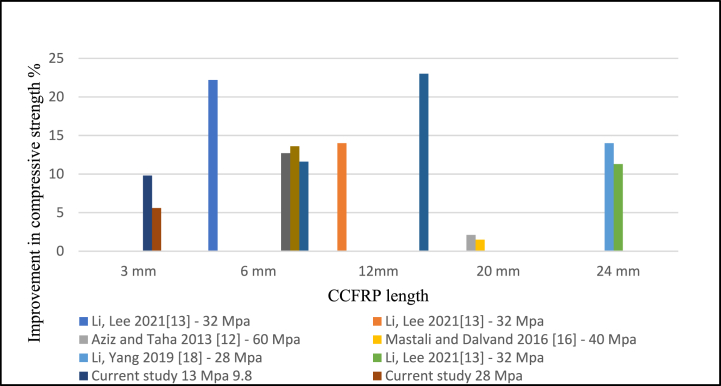
Compressive strength versus CCFRP length.

Based on the literature, the compressive strength of CCFRP concrete increased when 0.25% and 0.5% of CCFRP were added. In contrast, the current study revealed that the addition of 0.5% CCFRP to the concrete decreased its compressive strength. While the optimal ratio is 0.25% as shown in Fig. 15**.** The effects of fiber length on compressive strength were solely explored, and it was discovered that increasing fiber length leads to a better compressive result. However, more research is necessary to determine the impacts of fiber length on flexural and tensile strength Fig. 16.

**Fig. 17:**
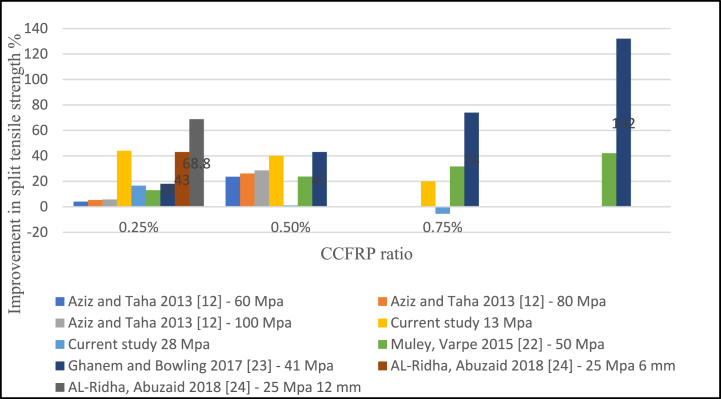
Split tensile strength versus CCFRP ratio.

**Fig. 18:**
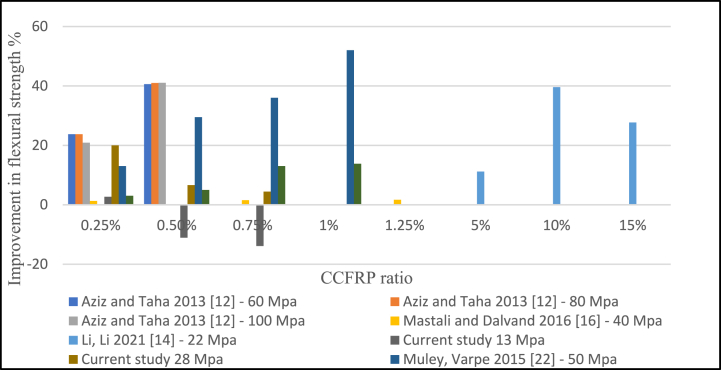
Flexural strength versus CCFRP ratio.

The Split tensile strength of concrete with CCFRP increased by 20–25% when only 0.5% CCFRP was added. Fig. 17 shows that 0.25% had less of an impact on the tensile strength of concrete. Based on the available data, a 0.25–0.5% CCFRP is recommended. Similarly, as illustrated in Fig. 18, the addition of 0.25% and 0.5% CCFRP increases flexural strength, however, increasing this dosage reduces flexural strength.

**Fig. 19:**
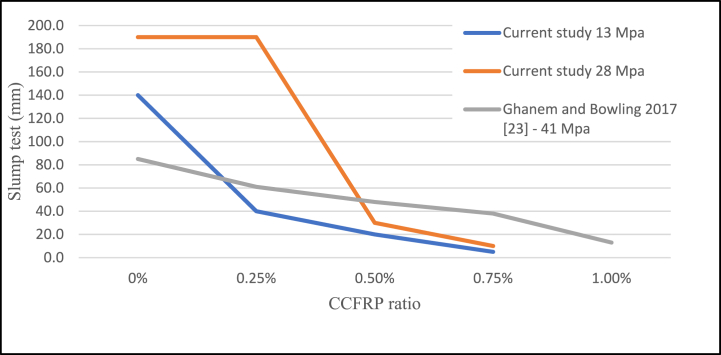
Slump test versus CCFRP ratio.

**Fig. 20:**
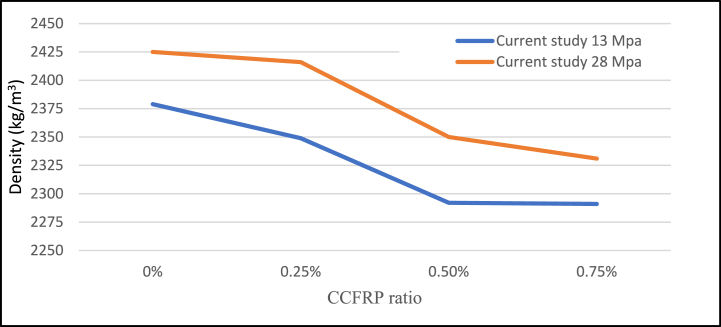
Density versus CCFRP ratio.

One of the biggest challenges with adding CCFRP to the concrete was workability, which is considerably altered by the presence of CCFRP. There is no date in the literature about slump test values if CCFRP is used. In this study, however, the slump test result is documented and shown in Fig. 19. To address this issue, a superplasticizer is used. The result showed that the optimal CCFRP ratio to regulate workability is 0.25% with a 14 to 19-cm slump test value, followed by 0.5%. Moreover, density was also recorded in this study as depicted in Fig. 20. The result suggested that the inclusion of CCFRP decreased the density of concrete.

## 5 Conclusion

Although the addition of CCFRP to the concrete mixture led to an improvement in concrete strength, it also had some undesirable side effects. The following are the principal findings of the study.

• The addition of CCFRP to the concrete mixture significantly decreased its workability.
• By adding CCFRP at a 0.25% optimum ratio, early age compressive strength of concrete enhanced from 15.1 MPa to 17.7 MPa which is about 17% for NSC and 18% for LSC.
• CCFRP raised the early-age split tensile strength of normal concrete from 2.5 MPa to 2.4 MPa, whereas low-strength samples increased by around 18% compared with the 1.8 MPa control sample.
• When 0.25% CCFRP was added to 28-day-old samples, their compressive strength improved dramatically. The boost was approximately 6% for normal strength samples compared with 28.5 MPa control samples and 10% for low strength samples compared with 13.2 MPa samples without fibers.
• Adding 0.25% CCFRP to the mix improves the split tensile strength of 28-day-aged samples by approximately 44% for NSC compared with 2.5 MPa control samples, and for LSC value increased from 1.8 MPa to 2.1 MPa which is about 17% enhancement.
• With the addition of 0.25% CCFRP, the flexural strength of samples of normal strength enhanced dramatically. Moreover, there was a minimal improvement with low-strength samples.
• CCFRP ratios of 0.5 and 0.75% lowered concrete compressive strength, split tensile strength, and flexural strength.

## 6 Recommendations and future direction

While adding chopped CFRP improved concrete strength significantly, it has certain downsides in terms of workability and doses detrimental effects. Furthermore, several aspects of this new material must also be investigated for future direction. The followings are the main recommendations and future work directions.

• The addition of chopped CFRP to concrete reduced its workability. As a result, water-reducer compounds are preferred for controlling workability.
• The impact of varied chopped CFRP lengths on concrete strength and workability must be investigated.
• The influence of additional chopped fiber materials on the mechanical characteristics of concrete must be investigated further. Materials such as Glass Fiber Reinforced Polymer (GFRP) and Basalt Fiber Reinforced Polymer (BFRP) can be obtained at a lower price than chopped CFRP.
• Use finite elements to create a model based on the result found in the experimental work.

## 7 List of notations

Table 11 illustrates the symbols and notations employed in this paper.

**Table 11:** List of notations and symbols.

Symbol	Description
ρb	The density of the cube in $\frac{kg}{m3}$.
M	Mass of the cube in kg.
V	The volume of the cube in m^3^.
*T*	Splitting tensile strength, MPa.
*P*	The maximum applied load indicated by the testing machine, N.
*D*	Diameter of the specimen, mm.
*L*	Length of the specimen, mm.
σ	Compressive strength of a cube, MPa.
A	Area of a loaded cube face, mm^2^.
*R*	Modulus of rupture, MPa.
*b*	The average width of the specimen, at the fracture, is mm.
*d*	The average depth of specimen, at the fracture, is mm.

## Author contribution statement

Mand Kamal Askar: Conceived and designed the experiments; Performed the experiments; Analyzed and interpreted the data; Contributed reagents, materials, analysis tools or data; Wrote the paper.Lawend K. Askar: Performed the experiments; Analyzed and interpreted the data.Yaman S. S. Al-Kamaki: Performed the experiments; Wrote the paper.Razaq Ferhadi: Contributed reagents, materials, analysis tools or data.

## Data availability statement

Data will be made available on request.

## Declaration of competing interest

The authors declare that they have no known competing financial interests or personal relationships that could have appeared to influence the work reported in this paper.
